# Impact of Inhaled Corticosteroids on the Modulation of Respiratory Defensive Reflexes During Artificial Limb Exercise in Ovalbumin-Sensitized Rabbits

**DOI:** 10.3389/fphys.2021.804577

**Published:** 2022-01-25

**Authors:** Sarah Basin, Simon Valentin, Silvia Demoulin-Alexikova, Bruno Demoulin, Laurent Foucaud, Delphine Gérard, Celso Pouget, Edem Allado, Bruno Chenuel, Mathias Poussel

**Affiliations:** ^1^Department of Pneumology, CHRU Nancy, Nancy, France; ^2^EA 3450 DevAH—Development, Adaptation and Disadvantage, Cardiorespiratory Regulations and Motor Control, Université de Lorraine, Nancy, France; ^3^Department of Pediatric Respiratory Function Testing, CHRU Nancy, Nancy, France; ^4^Laboratory of Hematology, CHRU Nancy, Nancy, France; ^5^Department of Pathology CHRU Nancy, Nancy, France; ^6^Pulmonary Function Testing and Exercise Physiology, CHRU Nancy, Nancy, France

**Keywords:** asthma, cough, exercise, corticosteroids (CS), defensive reflex

## Abstract

**Introduction:**

Cough is a major lower airway defense mechanism that can be triggered by exercise in asthma patients. Studies on cough reflex in experimental animal models revealed a decrease of cough reflex sensitivity during exercise in healthy animals, but a lack of desensitization in ovalbumin-sensitized rabbits. The aim of our study is to evaluate the impact of inhaled corticosteroids on cough reflex during artificial limb exercise in an animal model of eosinophilic airway inflammation.

**Materials and Methods:**

Sixteen adult ovalbumin-sensitized rabbits were randomly divided into two groups. The “OVA-Corticoid” group (*n* = 8) received inhaled corticosteroids (budesonide; 1 mg/day during 2 consecutive days) while the “OVA-Control” (*n* = 8) group was exposed to saline nebulization. The sensitivity of defensive reflexes induced by direct mechanical stimulation of the trachea was studied in anesthetized animals, at rest and during artificial limb exercise. Cell count was performed on bronchoalveolar lavage fluid and middle lobe tissue sections to assess the level of eosinophilic inflammation.

**Results:**

All rabbits were significantly sensitized but there was no difference in eosinophilic inflammation on bronchoalveolar lavage or tissue sections between the two groups. Artificial limb exercise resulted in a significant (*p* = 0.002) increase in minute ventilation by 30% (+ 209 mL.min^–1^, ± 102 mL/min^–1^), with no difference between the two groups. 322 mechanical tracheal stimulations were performed, 131 during exercise (40.7%) and 191 at rest (59.3%). Cough reflex was the main response encountered (46.9%), with a significant increase in cough reflex threshold during artificial limb exercise in the “OVA-Corticoid” group (*p* = 0.039). Cough reflex threshold remained unchanged in the “OVA-Control” group (*p* = 0.109).

**Conclusion:**

Inhaled corticosteroids are able to restore desensitization of the cough reflex during artificial limb exercise in an animal model of airway eosinophilic inflammation. Airway inflammation thus appears to be involved in the physiopathology of exercise-induced cough in this ovalbumin sensitized rabbit model. Inhaled anti-inflammatory treatments could have potential benefit for the management of exercise-induced cough in asthma patients.

## Introduction

Cough is a physiological respiratory defense mechanism regulated by a complex reflex arc to protect the lower airways. However, when cough becomes chronic (lasting more than 8 weeks), it may directly impact patient’s quality of life (Chamberlain et al., 2015). Chronic cough can be associated with various underlying disorders, including chronic obstructive pulmonary disease or gastro-esophageal reflux, but diseases involving eosinophilic airway inflammation such as asthma appear to be predominant (Diver et al., 2019). Exercise is a common trigger of cough in asthma patients, prevalence studies have shown that up to 80% of untreated asthma patients may experience an exercise-induced exacerbation of their symptoms, including coughing (Del Giacco et al., 2015). The pathophysiological mechanisms involved in exercise-induced cough are multiple and complex, and its management is not currently standardized. Recent international recommendations from the Global Initiative for Asthma (*GINA*) suggest rescue therapy in the event of symptom onset or before exercise, combining a low-dose inhaled corticosteroid and a long-acting bronchodilator (GINA, 2020 report). These recommendations have not yet been adopted by French Society of Respiratory Disease, which recommends the use of a short-acting bronchodilator in these situations (Recommandations pour la prise en charge et le suivi des patients asthmatiques, 2020).

However, there is evidence to suggest that when cough stimulation is imposed during exercise, the respiratory centers focus on adapting to the metabolic needs imposed by exercise, with an increase in ventilation and a decrease in cough sensitivity (Kondo et al., 1998; Lavorini et al., 2010). Previous study conducted on anaesthetized rabbits supports this hypothesis, with an increase in the cough reflex threshold during exercise (Poussel et al., 2014). On the opposite, in an animal model of eosinophilic airway inflammation (ovalbumin-sensitized rabbits), there was an absence of desensitization of the cough reflex during artificial limb exercise suggesting the key role of inflammation in the occurrence of exercise-induced cough (Tiotiu et al., 2017). Intravenous corticosteroids appear to be able to restore the desensitization of the cough reflex during artificial limb exercise in the same experimental animal model (Valentin et al., 2020). The main goal of this study was to investigate the impact of inhaled corticosteroid therapy on exercise-induced respiratory defense reflexes in ovalbumin-sensitized rabbits.

## Materials and Methods

### Ethical Approval

The study involved sixteen New Zealand adult rabbits (8 females, weight = 3.09 kg ± 0.12) housed in a conventional facility at Animal House of the Campus Biology-Health, University of Lorraine, from February to April 2021. The experimental procedures were developed in accordance with the local ethics committee on animal testing. The study protocol was approved by the Comité d’Éthique Lorrain en Matière d’Expérimentation Animale (CELMEA) and the *Ministère de l’Enseignement Supérieur, de la Recherche et de l’Innovation* (APAFIS #26735). Animal housing and experiments were performed according to the directive 2010/63/EU of the European Parliament and of the council of 22 September 2010 on the protection of animals used for scientific purposes. Principle of replacement, reduction and refinement were ensured in order to reduce to the minimum not only the number of animals used but also any possible pain, suffering, distress or lasting harm to the animals. Animal suppliers were authorized by, and registered with the competent authority. All animals were provided with appropriate accommodation, environment, food, water and care to their health and well-being. Rabbits were randomized in two groups: “OVA-Corticoid” group (*n* = 8) and “OVA-Control” group (*n* = 8).

### Sensitization Protocol

**FIGURE 1:**
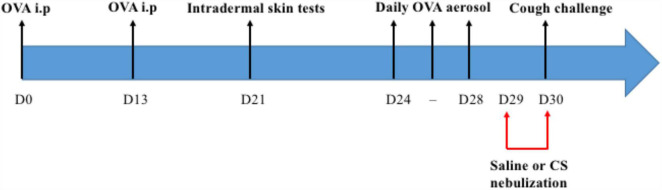
Pre-experimental interventions. Each rabbit received intraperitoneal (i.p) injections of ovalbumin (OVA), an intradermal skin test and a daily aerosol of OVA for 3 consecutive days between D24 and D28. They were challenged with saline or corticosteroid (CS) nebulizations at D29 and D30.

All rabbits were sensitized to ovalbumin by two peritoneal injections on day 0 and day 13 of a solution containing 1 mL of saline (0.9% NaCl) in which 0.1 mg of ovalbumin and 10 mg of aluminum hydroxide Al(OH)^3–^ were dissolved. Ten days later, rabbits were exposed daily for 3 days to aerosols of ovalbumin at a concentration of 2.5 mg/L (50 mg of ovalbumin dissolved in 20 mL of 0.9% NaCl). Each aerosol was administered in a Plexiglas box using an ultrasonic nebulizer (SYST’AM, LS290) producing droplets with an aerodynamic diameter between 1 and 5 μm for 20 min per nebulization. The last OVA aerosol was performed 48 h before the mechanical cough challenge (Figure 1). Sensitization was verified by performing intradermal skin tests at day 21 with subcutaneous injection of 0.1 mL of a 200 μg/L ovalbumin solution into the skin of the dorsal region of each rabbit. A saline injection was performed following the same protocol (0.1 mL in the dorsal region). The reaction was assessed at 24 h by measuring the induration at the injection site using an electronic caliper (RS Pro Electronic Digital Caliper).

### Corticosteroid Nebulization

Rabbits randomized to the “OVA-Corticoid” group received two corticosteroid nebulization (one mg of budesonide dissolved in 20 mL of saline) the day before and 1 h before the cough challenge. Each aerosol was administered for 20 min, using the device described above (Plexiglas box with an ultrasonic nebulizer). Rabbits randomized to the “OVA-Control” group were exposed to saline aerosol, following the same experimental design (i.e., 24 and 1 h before the experiment) (Figure 1).

### Experimentation

Analgesia premedication was performed by an intramuscular injection of buprenorphine 20 μg/kg (Bupaq multidose 0.3 mg/mL) in hind limb muscle. Induction of anesthesia was conducted 15 min after premedication by a slow intravenous injection over 1 min, in the marginal vein of the ear, of a solution of propofol (Propovet) 3 mg/kg and ketamine 3 mg/kg. As soon as induction was complete, maintenance of anesthesia was initiated with propofol 0.8 mg/kg/min and ketamine 0.2 mg/kg/min, administered *via* the catheter left in place (i.e., marginal vein of the ear). The depth of anesthesia was monitored throughout the experiment by respiratory rate, the presence of a corneal reflex but the absence of ear retraction and ear pinch response. At the end of the experiment, euthanasia was performed by an intravenous injection of pentobarbital (Exagon) 1 mL/kg (i.e., 400 mg/kg).

A sagittal midline incision of the skin tissues of the neck was made on anesthetized rabbit, followed by careful dissection of the subcutaneous and muscular tissues. The trachea was partially transected transversely and each rabbit was tracheostomized (at the caudal part of the trachea) and intubated using a steel tracheostomy cannula, allowing spontaneous breathing. Body temperature was recorded continuously with an electronic thermometer (Physiotemp Instruments, YSI 402 Clifton, NJ, United States) and maintained at 38 °C using circulating warm water pad. Heart rate was monitored by electrocardiogram electrodes placed on the chest. Tidal volume (VT), respiratory frequency (RF) and ventilated flow were recorded throughout the entire experiment by a pneumotachograph (No. 0 Fleisch pneumotachograph with linear range ± 250 mL/s) connected to the tracheal cannula. As described previously, airway resistance (Rrs) was measured by adaptation of the forced oscillation technique to assess exercise-induced bronchodilation (Poussel et al., 2014).

The exercise simulation protocol on anesthetized rabbits consisted of electrical stimulation of hind limb muscles by electrodes (Dura-Stick Premium, REF 42205, DJO, United States) connected to an electrical stimulator (Neuro Trac Rehab, Verity Medical LTD, United Kingdom). Muscle contractions were triggered by a 2 s electrical stimulation with an intensity between 10 and 30 mA. Each stimulation was separated by a free interval of 4 s. The protocol was maintained for 4 min, in order to increase ventilation by at least 30% compared to resting ventilation. Respiratory variables were recorded at rest and during muscle contractions induced by muscle electrostimulation.

A semi-rigid Silastic^®^ catheter (0.7 mm, OD Metric) connected to a rotary electric motor (low voltage DC motors 719RE280, MFA/Comodrills, United Kingdom) was inserted into the tracheostomy cannula to stimulate the airway mucosa and trigger a respiratory defensive reflex. The electric motor has a rotating speed of about 60 revolutions per minute allowing mechanical tracheal stimulations of 50–1,000 ms. All stimulations were delivered during inspiratory phase in order to promote the cough reflex over the expiratory reflex (Varechova et al., 2010). Three sequences of four stimulations were performed at rest and during artificial limb exercise on each rabbit with 10 min of recovery without any stimulation between each sequence.

The electromyographic activity of abdominal muscles was measured by insertion of bipolar stainless steel wire electrodes (A-M Systems INC, Sequim, WA 98,382) in either the transverse abdominal muscles or the external oblique muscles in order to confirm active respiratory movement associated with the response to tracheal stimulation.

### Bronchoalveolar Lavage

After tracheal stimulation, a polyethylene-190 catheter was positioned *via* the endotracheal cannula at the carina and lavage was performed by slow injection followed by aspiration of 5 mL of saline 3 times. Bronchoalveolar Lavage (BAL) was collected and filtered with a nylon tissue perforated with 60 μm mesh to remove mucus. The solution was then diluted (1/10), placed in a centrifuge for 10 min at a speed of 600 rpm (Cytospin AutoSmear OF-120E) and stained by the May-Grunwald-Giemsa technique to identify cell populations. On each slide, at least 100 cells were counted using light microscopy (Olympus CDD camera in 1,360 × 1,024 pixel, ×40 objective) and differentiated according to their morphological characteristics between: macrophages, lymphocytes, neutrophils, eosinophils, basophils and monocytes.

### Histology

Following bronchoalveolar lavage and animal euthanasia, a median sternotomy was performed to collect the middle lobe of the right lung of each rabbit and a sample of trachea, at distance from the tracheotomy orifice. All specimens were 4% formalin fixed and paraffin embedded. Sections of 2 to 4 μm thickness were carried out with a microtome and then stained with a trichromatic hematoxylin eosin saffron stain. For each rabbit, a pathologist selected the area that appeared to contain the greatest number of inflammatory elements in the trachea and lung. A cell count of eosinophils under light microscopy was performed on the selected area and on 10 consecutive fields at ×400 magnification. The results were expressed as the mean number of eosinophilic cells per field for each rabbit, at the pulmonary and tracheal level.

### Data Analysis

Analogic signals were acquired using a PowerLab 30 series system (ADInstruments, ML880 PowerLab 16/30) with an acquisition frequency of 200 Hz and a sampling resolution of 16 bits. The digitized data was analyzed using LabChart8-Pro software (ADInstruments, v 7.1). Before cough challenge, baseline values (at rest and during artificial limb exercise) were recorded by averaging the respiratory variables (VT, RF, $\dot{\text{V}}$Emax: peak expiratory flow) over three consecutive respiratory cycles. The response to tracheal stimulation was assessed by changes in VT and $\dot{\text{V}}$Emax compared to baseline values. For each of the parameters, standard deviation (SD) was also calculated, providing information on the variabilitý of these parameters during spontaneous ventilation. A significant defensive response to mechanical tracheal stimulation was defined by a response in which the ventilatory parameters considered were outside the 99th percentile (i.e., above the mean + 3 times SD). Cough reflex was defined by a significant increase in VT, peak expiratory flow ($\dot{\text{V}}$Emax) and EMG. The cough threshold (CT) was defined as the shortest stimulation duration necessary to provoke at least one cough reflex. Expiratory reflex was retained in case of an isolated increase in $\dot{\text{V}}$ Emax.

Statistical analysis was performed with JMP 9.0.0 software (2010 SAS Institute Inc.) allowing comparisons of qualitative and quantitative data. The incidence and analysis of responses to mechanical tracheal stimulation in both conditions (rest and artificial limb exercise) were analyzed by the Chi-square (χ^2^) or Fisher test. Paired *t*-test was used for the comparison of respiratory variables (VT, $\dot{\text{V}}$E, Rsr) and cell counts in the lung and trachea and Mann-Whitney *U*-test was used for the comparison of cell counts in BAL. Wilcoxon non-parametric test was applied to compare rest and exercise cough threshold. Results are expressed as mean ± SD and a statistical significance was retained for a *p* < 0.05. The primary endpoint was the cough reflex threshold (in milliseconds).

## Results

### Population

All 16 rabbits completed the ovalbumin sensitization protocol. There was no significant difference between the two groups in terms of sex and weight (“OVA-Control” group: 3.11 ± 0.10 kg; “OVA-Corticoid” group: 3.07 ± 0.13 kg, *p* = NS). A death occurred during induction of anesthesia in the “OVA-Control” group, the rabbit was excluded from the study as no data regarding respiratory defense reflexes could be obtained.

### Ventilation and Reflexes

The exercise simulation protocol resulted in an increase in minute ventilation in both groups, from 680 mL.min^–1^ ± 128 mL/min^–1^ at rest to 889 mL/min^–1^ ± 128 mL/min^–1^ during exercise (+ 30.7%, *p* = 0.002; paired *t*-test). Overall, Rrs decreased significantly from 20.4 ± 4.39 hPa.s.L^–1^ to 17.2 ± 3.32 hPa.s.L^–1^ (*p* = 0.011; paired *t*-test) during muscle contractions. There was no significant difference in minute ventilation and respiratory resistance at rest and during artificial limb exercise between the two groups (*p* = NS; paired *t*-test).

**FIGURE 2:**
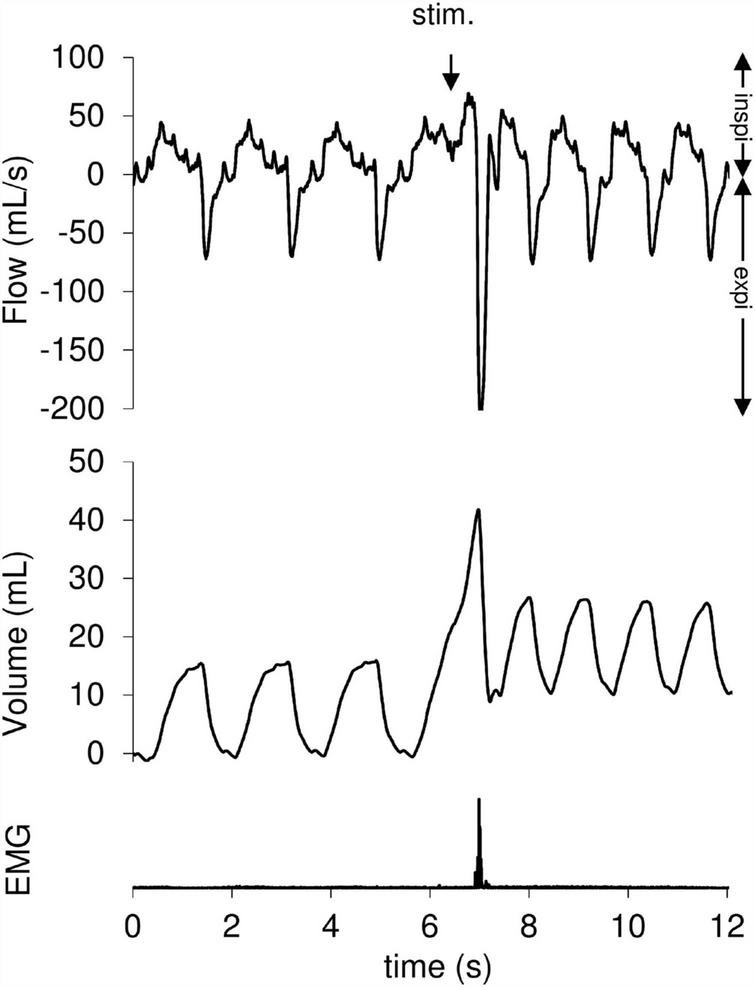
Cough reflex following a tracheal mechanical stimulation. The cough reflex is characterized by an increase in tidal volume (V_T_) associated with a concomitant increase of peak expiratory flow ($\dot{\text{V}}$ Emax). The downward arrow indicates tracheal stimulation (Stim.). Positive and negative airflow rates indicate inspiration (insp.) and expiration (exp.), respectively. Abdominal muscles electromyogram (EMG) also showed activity on the stimulation breath.

During the experiment, 322 mechanical tracheal stimulations were performed, 131 during artificial limb exercise (40.7%) and 191 at rest (59.3%). The responses obtained after tracheal stimulation were an expiratory reflex in 17.1% of cases, a cough reflex in 46.9% of cases (Figure 2) and no response in 36% of cases. The cough reflex threshold in milliseconds was measured for each rabbit (Table 1). There was a significant increase in the exercise cough reflex threshold in the “OVA-Corticoid” group (*p* = 0.039; Wilcoxon) representing a restoration of cough reflex desensitization during artificial limb exercise in these sensitized rabbits treated with inhaled corticosteroids. No change in cough reflex threshold during muscular electrostimulation was found in the “OVA-Control” group (*p* = 0.109; Wilcoxon).

**TABLE 1:** Distribution of cough reflex (CR) threshold (expressed in milliseconds) at rest and during exercise in both groups (“OVA-Corticoid” group and “OVA-Control” group).

« OVA-Corticoid » group			« OVA-Control » group		
Rabbit	CR threshold at rest	CR threshold during exercise	Rabbit	CR threshold at rest	CR threshold during exercise
5	150	600	1	1,000	1,000
6	300	300	2	50	50
7	150	150	3	150	150
8	50	50	11	600	300
9	50	300	12	300	150
10	50	150	13	300	50
15	50	150	14	150	150
16	50	150			

*CR threshold increased during exercise in the “OVA-Corticoid” group (p = 0.039; Wilcoxon test) whereas it was unchanged in the “OVA-Control” group (p = 0.109; Wilcoxon test).*

### Bronchoalveolar Lavage

Eosinophils were the predominant inflammatory cells found in BAL, representing 13.7% of the cells in the “OVA-Control” group compared to 12.3% in the “OVA-Corticoid” group (*p* = NS; Mann-Whitney *U*-test). There was no difference in eosinophilic or neutrophilic inflammation in the bronchoalveolar lavage fluid between the two groups (Table 2).

**TABLE 2:** Mean cell counts of eosinophils and neutrophils in bronchoalveolar lavage (expressed as% of total cell count) in both groups (“OVA-Corticoids” group and “OVA-Control” group).

	« OVA-Corticoid » group	« OVA-Control » group	*p*
Eosinophils (%)	12.3	13.7	NS
Neutrophils (%)	0.12	0.71	NS

*Mann-Whitney U-test for comparison between groups.*

### Histology

Eosinophilic inflammation was significantly more important in parenchymal than in tracheal areas in the “OVA-Control” group (*p* < 0.01, *p* = NS in “OVA-Corticoid” group; paired *t*-test). There was no difference in tracheal eosinophilic infiltration between the two groups (*p* = NS; paired *t*-test; Table 3). A trend toward a decrease in eosinophilic inflammation in the lung was found in the “OVA-Corticoid” group (*p* = 0.07; paired *t*-test).

**TABLE 3:** Tracheal and parenchymal eosinophil cell counts expressed as number of cells per field (mean ± standard deviation) in both groups (“OVA-Corticoids” group and “OVA-Control” group).

	« OVA-Corticoid » group	« OVA-Control » group	*P*
Trachea	9.79 ± 6.5	11.1 ± 8.6	NS
Lung	13.1 ± 5.3	15.8 ± 8.4	NS (0,07)

*Paired t-test for comparison between groups.*

## Discussion

This study is the first to evaluate the impact of inhaled corticoids on cough reflex sensitivity during artificial limb exercise in a model of allergic airway inflammation. Corticosteroids appear to be able to increase cough reflex threshold during exercise. Despite the lack of some more control groups such as non-exercise animals with or without ovalbumin, our finding highlights the key role of eosinophilic inflammation in the modulation of cough.

Our animal model (*Oryctolagus cuniculus*) is of particular interest to study the impact of inhaled cortisteroids on cough reflex *in vivo*. Indeed, the airway innervation of lagomorphs is close to humans with a similar organization of the cough reflex, including A δ and C fibers (Spina et al., 1998). Besides, the OVA-sensitized rabbit is a well-established model of asthmatic disease with a similar therapeutic response to corticoids to that observed in humans (Keir and Page, 2008). This model allowed us to perform a mechanical cough challenge in spontaneous ventilation under anesthesia unlike smaller animal models such as the guinea pig. All tracheal mechanical stimulations were carried out during the inspiratory phase to favor the cough reflex over the expiratory reflex (46.9% of CR against 17.1% of ER) (Varechova et al., 2010). However, general anesthesia results in decreased conductivity of neural pathways and inhibition of the central nervous system. Our model did not allow us to investigate the cortical modulation of the cough reflex nor its behavioral component (Plevkova et al., 2021).

Our original protocol of electrical muscle stimulation lead to a significant increase in minute ventilation in all rabbits, simulating a moderate effort. Exercise is a well-known cough trigger, especially in dry and cold environments (Turmel et al., 2012). Indeed, the hyperventilation imposed by exercise induces a significant heat and water loss responsible for mucosal edema and changes in bronchial caliber. In addition, it can cause epithelial damages and chronic airway inflammation (Kippelen and Anderson, 2012), with the release of inflammatory mediators such as prostaglandins able to stimulate cough receptors (Coleridge et al., 1976). In fact, airway inflammation appears to have a major impact on cough reflex modulation during exercise, involving medullary structures and higher brain areas, even if brainstem mechanisms underlying the cough reflex and its regulation are only partially understood (Mutolo, 2017; Cinelli et al., 2018). Indeed, an important degree of modulation of the CR has already been shown, involving inflammatory agents at various levels (peripheral and central) (Mutolo, 2017; Cinelli et al., 2018) either enhancing or decreasing CR. In healthy subjects, when stimulation is imposed during exercise, the respiratory centers appear to favor increased ventilation over cough reflex (Lavorini et al., 2010). In contrast, in an animal model of allergic airway inflammation, there appears to be a lack of desensitization of cough reflex during artificial limb exercise (Tiotiu et al., 2017). More precisely, eosinophilic inflammation seems to be particularly involved in the modulation of cough. Eosinophils can influence sensory pathways involved in defensive reflexes in numerous ways, including the release of extracellular ATP (Turner and Birring, 2019). Indeed, in OVA-sensitized mice exposed to allergen challenge, an increase ATP level was found in BAL fluid as in asthma patients (Idzko et al., 2007). Besides, the implication of the P2 × 3 receptors located on the terminal of C fibers in cough is now well-established (Bonvini and Belvisi, 2017). Inhalation of ATP is able to induce a chemical cough by stimulating these ligand-gated ion channels, but endogenous ATP released by inflammatory cells in the airways can also trigger these receptors (Turner and Birring, 2019). The efficiency of P2 × 3 receptor antagonists developed in refractory chronic cough demonstrates the major role of extracellular ATP in cough hypersensitivity syndrome (CHS) (Morice et al., 2019, 2021). Dedicated studies are needed to assess their relevance in exercise-induced cough.

In our study, we did not find a lower eosinophilic cell count in the parenchyma or BAL fluid in the “OVA-Corticoid” group compared to the “OVA-Control” group. This result could reflect an insufficient posology of budesonide although it corresponds to a high daily dose for an asthma patient (1,000 mg). In addition, the exact dosage of budesonide that reached the lower airways is uncertain due to the inhaled route. However, a functional impact of corticosteroids on eosinophilic cells with decreased release of inflammatory mediators cannot be excluded.

Despite the use of a well-established model of cough, our study has limitations. Allergic inflammation induced by sensitization to ovalbumin and aluminum hydroxide is not the most representative of asthma. Although this protocol allowed us an important eosinophilic airway inflammation, its impact on other key elements of the Th2 response potentially involved in cough such as mast cells or IL-4 receptors is less clear. For example, a sensitization to House Dust Mite, using only the inhalation route for sensitization and exposure to allergens, would be more in line with clinical reality (Buday et al., 2016). However, the superiority of this protocol over ovalbumin sensitization remains to be demonstrated. It is important to note that our exercise protocol was not able to significantly modify the airway resistances in either the “OVA-Corticoid” group or the “OVA-Control” group. Exercise-induced cough seems to be frequently related to bronchoconstriction, particularly in asthma patients, as smooth muscle contraction leads to SAR receptors stimulation (Widdicombe, 2001; Boulet and Turmel, 2019). However, according to other studies, the association between bronchomotricity and cough reflex sensitivity seems less clear (Lavorini et al., 2010). Finally, the use of buprenorphine, an opioid agonist-antagonist, may have an impact on cough sensitivity. Indeed, neuromodulators have proven antitussive properties and a daily low dose of morphine is currently recommended for the treatment of chronic refractory cough (Morice et al., 2007, 2020). Still, a single administration before the cough challenge should not have influenced our results.

## Conclusion

In conclusion, our study is the first to demonstrate the restoration of desensitization of cough reflex during artificial limb exercise by inhaled corticoids. Despite the lack of difference in inflammatory cell count, airway inflammation appears to be the key factor in the modulation of cough during exercise. Our results are thus in line with the recent *GINA* recommendations to use an inhaled corticoid before exercise in asthma patients. A better understanding of the complex mechanisms underlying exercise-induced cough might allow the development of an effective and much-needed therapy.

## Data Availability Statement

The original contributions presented in the study are included in the article/supplementary material, further inquiries can be directed to the corresponding author/s.

## Ethics Statement

The animal study was reviewed and approved by Comité d’Éthique Lorrain en Matière d’Expérimentation Animale and Ministère de l’Enseignement Supérieur, de la Recherche et de l’Innovation (APAFIS #26735).

## Author Contributions

SB, SV, SD-A, BC, and MP: study design and development, data analysis and interpretation, writing the manuscript, and final approval of the submitted version. BD and LF: study design and development, data analysis and interpretation, and final approval of the submitted version. DG, CP, and EA: study design and development, and final approval of the submitted version. All authors contributed to the article and approved the submitted version.

## Conflict of Interest

The authors declare that the research was conducted in the absence of any commercial or financial relationships that could be construed as a potential conflict of interest.

## Publisher’s Note

All claims expressed in this article are solely those of the authors and do not necessarily represent those of their affiliated organizations, or those of the publisher, the editors and the reviewers. Any product that may be evaluated in this article, or claim that may be made by its manufacturer, is not guaranteed or endorsed by the publisher.
